# Current situation of acute ST-segment elevation myocardial infarction in a county hospital chest pain center during an epidemic of novel coronavirus pneumonia

**DOI:** 10.1515/med-2022-0621

**Published:** 2023-01-09

**Authors:** Feng Li, Rong Luo, Xiao-Ting Wang, Jun-Feng Jia, Xue-Ying Yu

**Affiliations:** Department of Cardiology, Jieshou People’s Hospital, 339 Renmin Road, Jieshou, Fuyang, Anhui, 236500, China; Department of Cardiology, Jieshou People’s Hospital, Jieshou, Fuyang, Anhui, 236500, China

**Keywords:** myocardial infarction, COVID-19, primary hospitals, chest pain center, percutaneous coronary intervention

## Abstract

Our object was to examine how the pre- and post-pandemic COVID-19 impacted the care of acute ST-segment elevation myocardial infarction (STEMI) patients in county hospitals. Using January 20, 2020, as the time point for the control of a unique coronavirus pneumonia epidemic in Jieshou, 272 acute STEMI patients were separated into pre-epidemic (group A, *n* = 130) and epidemic (group B, *n* = 142). There were no significant differences between the two groups in terms of mode of arrival, symptom onset-to-first medical contact time, door-to-needle time, door-to-balloon time, maximum hypersensitive cardiac troponin I levels, and in-hospital adverse events (*P* > 0.05). Emergency percutaneous coronary intervention (PCI) was much less common in group B (57.7%) compared to group A (72.3%) (*P* = 0.012), and the proportion of reperfusion treatment with thrombolysis was 30.3% in group B compared to 13.1% in group A (*P* < 0.001). Logistic regression analysis showed that age ≥76 years, admission NT-proBNP levels ≥3,018 pg/ml, and combined cardiogenic shock were independent risk factors for death. Compared with thrombolytic therapy, emergency PCI treatment further reduced the risk of death in STEMI. In conclusion, the county hospitals treated more acute STEMI with thrombolysis during the COVID-19 outbreak.

## 1 Introduction

Acute myocardial infarction (AMI) has an acute onset, severe symptoms, and high mortality, and the death rate of AMI in China has been substantially higher in rural than in urban areas since 2013 [1]. In contrast to a general trend of diminishing AMI incidence and death in industrialized nations [2,3,4,5,6], it is estimated that the number of AMI cases in China would significantly grow from eight million in 2010 to 23 million in 2030 [7].

In the United States, Eberhard and colleagues found that the rate of heart disease mortality was greatest in the South, and rural regions had a rate 25% higher than Southern suburban people [8]. Rural and distant Australians have greater cardiovascular disease (CVD) risk factors, higher rates of CVD-related hospitalization, and are more likely to die from CVD than those in urban regions [9].

Early reperfusion therapy is an important measure for the salvage of acute STEMI patients, and early administration of fibrinolytic therapy and emergency primary percutaneous coronary intervention (PCI) can significantly reduce the mortality of ST-segment elevation myocardial infarction (STEMI) patients [10,11], and the earlier the reperfusion therapy within 12 h of onset, the higher the revascularization rate and the lower the in-hospital mortality [12]. Intravenous thrombolysis, primary angioplasty, intracoronary thrombolysis, or urgent coronary artery bypass grafting operations are examples of initial reperfusion treatment used to restore blood flow through a suspected or known occluded coronary artery shortly after diagnosis.

The choice of STEMI reperfusion treatment measures is influenced by the medical conditions, level of consultation and treatment, and geographical location in the area of consultation, and China Acute Myocardial Infarction studies have shown that the reperfusion ratio is lower in the county hospitals compared with provincial and municipal hospitals [13], but with the creation of chest pain centers in recent years, it has led to a significant improvement in the treatment of acute STEMI in the county hospitals, significantly shortened the rescue time, improved the reperfusion treatment ratio to varying degrees, and significantly improved the prognosis of STEMI patients [14,15]. Primary PCI is the recommended therapeutic method for patients with STEMI when done promptly, according to early randomized studies [16,17,18,19]. According to the most recent European Society of Cardiology recommendation, reperfusion treatment is advised in all STEMI patients with symptoms of ischemia lasting less than 12 h, and primary PCI is preferred over thrombolysis within the timeframes specified [20]. However, with the outbreak and rapid prevalence of the novel coronavirus pneumonia (COVID-19), the country adopted strict control measures for category A infectious diseases in order to control the rapid spread of the epidemic [21,22], taking measures such as travel restrictions, home isolation, and strengthening personal protection. The epidemic control and the psychological and emotional changes brought about by the epidemic, travel restrictions, and fear of hospital infection have affected the treatment of acute STEMI to some extent.

The impact of the COVID-19 pandemic on the management of acute STEMI patients has been reported differently in the domestic and international literature. Two domestic studies reported a decrease in the number of acute STEMI patient visits in high-risk areas, a delay in the symptom onset-to-first medical contact (S2FMC) time, a change in the choice of reperfusion therapy measures for acute STEMI patients, and prolongation of the door-to-balloon (D2B, interval between the patient’s arrival in the emergency department and ends when PCI with a catheter guidewire and balloon inflation crosses the culprit lesion) time during the pandemic [23,24]. Gramegna et al. demonstrate that patients with STEMI have a considerably longer duration from the beginning of symptoms to hospital admission when compared to the same timeperiod in the preceding two years [25], but no significant effect on prognosis has been found [23,24], and few relevant studies have been reported, mostly data from tertiary hospitals in high-risk areas during the pandemic period.

Increased mortality and worsening cardiac outcomes in AMI in the early phase of the epidemic [26]. A German study showed that the number of STEMI saved during the peak of the epidemic decreased significantly, but clinically important indicators such as preadmission ECG, D2B, and in-hospital mortality were not significantly affected, and the existing structure of the regionalized STEMI network ensured a high-quality standard of care [27]. In-hospital death rates were 15.2 versus 11.2% among patients with out-of-hospital STEMI plus COVID-19 versus out-of-hospital STEMI without COVID-19 (absolute difference, 4.1% [95% CI, 1.1−7.0%]; *P* = 0.007). In-hospital death rates were 78.5% versus 46.1% in patients with in-hospital STEMI plus COVID-19 versus in-hospital STEMI without COVID-19 (absolute difference, 32.4% [95% CI, 29.0−35.9%, *P* < 0.001) [28].

It is unclear how the COVID-19 pandemic affects the treatment of acute STEMI patients in the county hospitals in low-risk areas of the epidemic. There have been several divergent reports on hospital outcomes, particularly mortality, and cardiogenic shock [29,30,31]. This study aimed to explore the current situation and its impact on the treatment of acute STEMI patients in the chest pain centers of county hospitals before and after the COVID-19 pandemic.

## 2 Patients and methods

### 2.1 Study population

Inclusion criteria: 1. patients with STEMI, meeting the diagnostic criteria: ischemic chest pain or its equivalent, dynamic evolution of the AMI electrocardiogram (ECG), dynamic changes in serum myocardial injury marker levels (at least 2 markers); 2. two or more adjacent leads to ST-segment elevation (>0.2 mV in anterior leads and 0.1 mV in limb leads), and early reperfusion therapy within 12 h of the onset of the disease [10].

Exclusion criteria: 1. patients who refused to be hospitalized in our hospital after diagnosis; 2. onset time more than 12 h; 3. patients hospitalized for coronary artery disease with acute in-stent thrombosis resulting in acute STEMI; 4. COVID-19 patients.

### 2.2 Study method

This retrospective cohort research compared the management of STEMI at Jieshou county hospital chest pain center in China during the COVID-19 and pre-COVID-19 eras. Jieshou is in northwestern Anhui Province, China, covering an area of 667.3 km^2^, with a population of 834,000 (2019). According to the time point of January 20, 2020, the date of epidemic control in Jieshou City, STEMI patients admitted to Jieshou People’s Hospital from January 20, 2019 to January 20, 2020 were designated as the pre-epidemic group (group A, *n* = 130). STEMI patients admitted to Jieshou People’s Hospital from January 21, 2020 to January 20, 2021 were designated as the epidemic group (group B, *n* = 142). Patients’ age, gender, smoking history, history of hypertension, diabetes mellitus, dyslipidemia, new-onset atrial fibrillation, and comorbid other chronic diseases were collected, and patients’ arrival mode, type of infarction and choice of reperfusion therapy, S2FMC (median time), door-to-needle D2N (average time of admission to the emergency department to time that thrombolytics were given) time, door-to-balloon (D2B, median time) time, admission NT-proBNP levels, and highest hs-cTnI levels were retrospectively studied, in addition to thrombolytic recanalization rate, emergency PCI success rate, thrombolysis in myocardial infarction (TIMI) flow after emergency PCI, and multivessel lesions, major in-hospital adverse events, and length of stay.


**Ethics approval and consent to participate:** This study was approved by the Ethics Committee of Jieshou People’s Hospital (JSLC20210001). The study proceeded to conform to the ethical guidelines of the 1975 Declaration of Helsinki. All participants had signed the written consent forms.
**Consent for publication:** Written consents to publish the clinical information related to the participants were obtained.

## 3 Statistical analysis

SPSS 26.0 software was used to analyze the data. The measurement data that conformed to normal distribution was expressed as mean ± standard deviation (SD) with independent sample *t*-test analysis, and those not conforming to normal distribution was expressed as M(Q1, Q3) with Mann–Whitney test analysis. Count data were expressed as %, and the *χ* ^2^ test or fisher exact test was used for comparison between groups. The CUT off-bounds of the quantitative variables were calculated using the Receiver Operator Characteristic (ROC) curve, and the quantitative variables were converted to qualitative variables and included in the binary logistic regression analysis. The dependent variable was death, and the independent variables included age, male, new-onset atrial fibrillation, comorbid chronic disease, admission NT-proBNP, cardiogenic shock, and different treatment modalities. *P* < 0.05 was considered a statistically significant difference.

## 4 Results

### 4.1 General information

At baseline, there were no statistically significant differences in age, gender, hypertension, diabetes, dyslipidemia, new-onset atrial fibrillation, or combined other chronic illnesses between the two groups (*P* > 0.05). According to electrocardiogram finding, acute STEMI was characterized as anterior or inferior wall infarction, with no statistically significant difference in the kind of infarction between the two groups (*P* > 0.05) (Table 1).

**Table 1:** Comparison of baseline characteristics between groups (%, mean ± SD)

Index	Group A	Group B	*t*/*χ*²	*P* value
Number of cases	130	142		
Age (years)	64.96 ± 11.54	63.75 ± 12.80	0.820	0.420
Sex (male)	93 (71.5%)	108 (76.1%)	0.718	0.397
Smoking history	44 (33.8%)	48 (33.8%)	0.000	0.990
Hypertension	76 (58.5%)	68 (47.9%)	3.046	0.081
Diabetes mellitus	22 (16.9%)	31 (21.8%)	1.042	0.307
Hyperlipidemia	25 (19.2%)	24 (16.9%)	0.249	0.618
New onset atrial fibrillation	10 (7.7%)	6 (4.2%)	1.473	0.225
Other chronic diseases	33 (25.4%)	35 (24.6%)	0.020	0.889

STEMI: ST segment elevation myocardial infarction.

### 4.2 Comparison of STEMI patient visits between the two groups

The difference between S2FMC in group B and group A was not statistically significant (*P* > 0.05), but the proportion of thrombolysis after admission was significantly higher than that in group A (30.3 vs 13.1%), while the proportion of emergency PCI treatment was significantly lower than that in group A (57.7 vs 72.3%, *P* < 0.01, Table 2).

**Table 2:** Comparison of treatment situations between groups (%, Q1, Q3)

Index	Group A	Group B	*χ*²	*P* value
S2FMC time (min, median)	120 (60, 180)	120 (64.75, 202.25)	1.086	0.277
Hospital arrival method	130	142		
Self-attendance	88 (67.7%)	103 (72.5%)	1.103	0.85
First-aid Ambulance	37 (28.5%)	35 (24.6%)		
In-hospital onset	3 (2.3%)	2 (1.4%)		
Outpatient transfer	2 (1.5%)	2 (1.4%)		
Treatment	130	142	17.248	0.002
Thrombolysis	17 (13.1%)	43 (30.3%)	11.685	0.001
Emergency PCI	94 (72.3%)	82 (57.7%)	6.301	0.012
Emergency CAG	4 (3.1%)	7 (4.9%)	0.600	0.438
Elective	2 (1.5%)	5 (3.5%)	1.064	0.302
Conservative	13 (10%)	5 (3.5%)	3.82	0.051

STEMI, ST-segment elevation myocardial infarction; S2FMC, symptom onset-to-first medical contact; PCI, primary percutaneous coronary intervention; CAG, coronary angiography.

### 4.3 Comparison of reperfusion therapy in STEMI patients in the two groups

There were no statistically significant differences in reperfusion treatment, D2N time, thrombolysis recanalization rate, post-thrombolysis PCI rate, number of successful emergency PCI cases, D2B time, post-procedure TIMI flow grade III rate, and proportion of multivessel lesions between the two groups (Table 3).

**Table 3:** Comparison of reperfusion treatments between groups (%, Q1, Q3, mean ± SD)

Index	Group A	Group B	*Z*, *t*/*χ*²	*P* value
Reperfusion therapy	115 (88.5%)	132 (93%)	1.644	0.200
Thrombolytic therapy	17	43		
Recanalization rate	14 (82.4%)	32 (74.4%)	0.446	0.504
Post-thrombolysis PCI rate	9 (52.9%)	28 (65.1%)	0.764	0.382
Post-procedure TIMI flow grade III rate	7 (77.8%)	19 (67.9%)	0.334	0.563
D2N time	61.88 ± 40.32	40.98 ± 16.49	2.070	0.053
Proposed emergency PCI	98	89		
Number of successful emergency PCI cases	94 (95.92%)	82 (92.13%)	1.206	0.272
D2B time (median)	87 (68.75,110.5)	83.5 (70,107.5)	0.332	0.740
Number of stents implanted	1.24 ± 0.61	1.43 ± 0.80	1.807	0.073
Multivessel lesions	75 (79.7%)	60 (73.17%)	1.073	0.300
Post-procedure TIMI flow grade III rate	93 (98.9%)	82 (100%)		1.000

STEMI, ST segment elevation myocardial infarction; TIMI, thrombolysis in myocardial infarction; PCI, primary percutaneous coronary intervention; D2B, door to balloon; D2N, door to needle.

### 4.4 Comparison of cardiac function indexes and adverse events between two groups of STEMI patients

The admission NT-proBNP levels in group B was lower than that in group A, and the difference was statistically significant (*P* < 0.05), but the differences in bleeding, heart failure, cardiogenic shock, highest hs-cTnI (ng/ml) levels, adverse events and length of stay were not statistically significant compared with group A (*P* > 0.05, Table 4).

**Table 4:** Comparison of heart function parameters and adverse events between groups (%, Q1, Q3)

Index	Group A	Group B	Z/*χ*²	*P* value
Admission NT-proBNP (pg/ml)	392 (123, 1,628)	193 (70.5, 944)	2.455	0.014
Highest hs-cTnI (ng/ml)	4.9 (0.32, 25.1)	4.13 (0.56, 24.93)	0.375	0.708
Total adverse cardiovascular events	32 (24.6%)	28 (19.7%)	0.947	0.331
Bleeding	2 (1.5%)	8 (5.6%)	3.463	0.063
Heart failure	15 (11.5%)	19 (13.4%)	0.210	0.646
Malignant arrhythmia	15 (11.5%)	10 (7.0%)	1.644	0.200
Cardiogenic shock	16 (12.3%)	19 (13.4%)	0.070	0.792
Death	15 (11.5%)	12 (8.5%)	0.724	0.395
Length of hospitalization (days)	6.34 ± 3	6.57 ± 3.4	0.598	0.550

STEMI, ST-segment elevation myocardial infarction; NT-proBNP, N-terminal pro-brain natriuretic peptide; hs-cTnI, hypersensitive cardiac troponin I.

### 4.5 Binary logistic regression analysis of the risk of death in STEMI patients

A binary logistic regression analysis with whether the death was the dependent variable and age, male, new-onset atrial fibrillation, comorbid chronic disease, positive admission NT-proBNP, cardiogenic shock, and different treatment modality factors as independent variables revealed that age ≥76 years, admission NT-proBNP levels ≥3,018 pg/ml and comorbid cardiogenic shock were independent risk factors for death in primary care STEMI patients.

Compared with thrombolytic therapy, emergency PCI further reduced the risk of death in older adults (76 years and older) with high NT-proBNP levels (where age and admission NT-proBNP levels were divided into two groups using ROC cutoff values of patients’ age of 76 years and admission NT-proBNP levels of 3,018 pg/ml, and treatment modalities were divided into three groups: thrombolysis, emergency PCI and others, Table 5).

**Table 5:** Logistic regression analysis of mortality risks in STEMI patients

Variables	B	SE	Wald	*P* value	OR	95% CI	
						Lower bond	Upper bond
Cardiogenic shock	2.376	0.666	12.726	0	10.761	2.917	39.696
Admission NT-proBNP	1.883	0.676	7.745	0.005	6.571	1.745	24.743
Age ≥76 years	1.336	0.664	4.052	0.044	3.804	1.036	13.973
Treatment modality			14.438	0.001			
Treatment modality (emergency PCI)	−2.04	0.756	7.282	0.007	0.13	0.03	0.572
Treatment modality (other)	0.907	0.738	1.513	0.219	2.477	0.584	10.517

STEMI, ST-segment elevation myocardial infarction; PCI, primary percutaneous coronary intervention; OR, odds ratio; CI, confidence interval; NT-proBNP, N-terminal pro-brain natriuretic peptide.

## 5 Discussion

With the popularization of the core concept of “time is myocardium, time is life” in primary hospitals and the guidance of acute STEMI guidelines [10], more and more primary hospitals have created primary or standard versions of chest pain centers, which standardize the diagnosis and treatment process of acute STEMI patients and improve the efficiency and shorten the total ischemic time, diagnosis and treatment process, improving the efficiency of treatment, shortening the total ischemic time, and significantly improve the prognosis of STEMI patients [32]. The COVID-19 outbreak lockdown, including access restrictions and traffic restrictions, was the primary prevention and control measure taken early on in most parts of China, which will inevitably have an impact on the emergency care of acute STEMI patients.

The results of this study found no statistically significant differences yet in the number of admissions and S2FMC in primary hospital chest pain centers, unlike earlier national and international studies. Data from Hangzhou, China, showed that the S2FMC time, D2B time, and catheterization laboratory activation time were significantly longer during the COVID-19 epidemic than before the epidemic, with a higher cumulative mortality rate at 28 days after surgery and longer inpatient consultation time [24]; the international studies on the impact of the COVID-19 epidemic on the treatment time of STEMI patients found a significant delay in the S2FMC time [33,34,35]. The reason for this may be the delay in going to the hospital for fear of infection during the pandemic.

The avoidance of medical care by ACS patients has led to severe clinical presentations and poor prognosis [36]. Delayed calls to the emergency medical services (EMS) system in STEMI patients despite having developed symptoms of myocardial infarction, with the time between symptom onset and call to the EMS system being three times longer than before the epidemic, is also the main reason for the delay between symptom onset and PPCI [37]. In addition, the post-visit epidemiological collection and protective measures in the emergency room and catheterization laboratory also contributed to a certain extent to the delay in treatment.

There was no difference in the way STEMI patients came to the hospital in low-risk areas during the epidemic compared with the pre-epidemic period, and the mode of access to the hospital was still dominated by self-access, with only 24.6% choosing ambulance access, indicating that although the awareness of “call an ambulance for chest pain” has been popularized to some extent in the chest pain centers of primary hospitals, most patients are reluctant to choose ambulance consultation due to the low cultural and economic level of the primary group and the lack of awareness of the disease, which is also an important reason for the delay in the treatment of acute STEMI [38]. The proportion of patients who chose thrombolytic therapy during the epidemic increased significantly and the proportion of emergency PCI decreased, with no statistically significant differences between the two groups in terms of adverse events such as bleeding complications, heart failure, shock, malignant arrhythmias, and death. These are in agreement with the study by Zhu et al. [39]. Emergency PCI is currently recognized by guidelines as the most effective life-saving measure for patients with acute STEMI. However, in recent years, an increasing number of studies on the efficacy and prognostic impact of early PCI after thrombolysis compared with emergency PCI have shown that thrombolysis combined with early PCI during an epidemic can further reduce the duration of myocardial ischemia in patients with acute STEMI, and thrombolysis did not increase poor clinical outcomes compared with emergency PCI [40].

It was shown that a strategy of thrombolysis-first during the epidemic was associated with lower rates of timely coronary reperfusion and increased incidence of recurrent ischemia and cardiogenic shock and exacerbated heart failure, but there was no increase in primary endpoint events such as composite death compared with 2019 [41]. Further studies of specific measures of reperfusion therapy found no differences between reperfusion therapy rates, thrombolytic recanalization rates, post-thrombolysis PCI rate, emergency PCI success rates, number of stents implanted, D2N time, D2B time, post-procedure TIMI flow grade III rate, and percentage of multivessel lesions in acute STEMI patients within 12 h of onset in the county hospitals during the epidemic. The reperfusion levels in our acute STEMI patients were slightly higher than the mean reperfusion levels of 83.2% in the 2020 Chest Pain Center Quality Control Report [42], and the D2B time met the requirement of less than 90 min chest pain center, but the D2N time was a little delayed from the chest pain center requirement within 30 min, but the overall trend was continuous improvement.

The first-to-treat principle was followed during COVID-19, and the emergency PCI process was the same as it was prior to the epidemic because we were in a low-risk area, so the D2B and S2FMC were not prolonged. Our results showed that the D2N time was shorter during the epidemic due to the priority given to thrombolysis during the epidemic, which shortened the communication time with the patients. Furthermore, during this time, the patient’s travel time to our hospital was not considerably impacted; hence, the findings of this study did not lead to a major change in S2FMC time. It is important to note that the majority of patients at primary care facilities have relatively low literacy and socioeconomic levels. Additionally, the first-choice STEMI treatment method during an outbreak, thrombolysis, is quite affordable. The increased rate of reperfusion during the epidemic was attributed to an increased rate of thrombolysis compared to emergency PCI [43], and the impact of the COVID-19 epidemic did not result in more severe adverse cardiovascular outcomes in primary care county hospitals that followed guidelines and consensus for the management of acute STEMI patients under a strict form of epidemic prevention and control during the COVID-19 epidemic.

Under the normalized epidemic prevention and control, improving the emergency PCI treatment rate and level is an important measure to reduce the mortality rate of STEMI patients in county hospitals. Therefore, under the normalized epidemic prevention and control, the chest pain center of the county hospital should still increase the publicity of AMI-related knowledge, improve the public’s awareness of AMI emergency treatment, shorten the treatment time, and improve the emergency PCI treatment rate and level is a key measure to reduce the mortality of acute STEMI patients. Because the initial stage in the STEMI chain of events is the identification of symptoms and rapid activation of the 9–1–1 system, community participation is critical. The difficulties of living in the country are self-evident. Ambulance staffing and crew training are recurring difficulties in the prehospital context, where EMS employees are frequently volunteers. It’s less probable that the providers are paramedics; as a result, sophisticated life support may not be widely available [44]. In this study, the mortality rate of acute STEMI patients was reported as 8.5%, which was significantly higher than the average in-hospital mortality rate of 3.77% for STEMI patients mentioned in the quality control report of the Chest Pain Center in 2020 [42], and logistic regression analysis found that patients’ age ≥76 years, admission NT-proBNP levels ≥3,018 pg/ml and combined cardiogenic shock were independent risk factors. Compared with thrombolytic therapy, emergency PCI treatment further reduced the risk of death.

The reasons for the high mortality rate of patients with acute STEMI in primary hospitals are considered as follows: 1. The success rate of emergency PCI in the county hospitals is lower than that in tertiary hospitals, due to the complexity of the lesion and the limitation of the technical level of the personnel performing emergency PCI to successfully open the vessel, which delays the rescue time and may cause a poor prognosis. 2. The level of reperfusion therapy is still a certain gap compared to tertiary hospitals, and the rate of emergency PCI is lower [45], the post-thrombolysis PCI rate is low, the average level in this study is 61.7%, according to the guidelines after thrombolysis all patients are recommended to perform early interventional intervention within 2–24 h or remedial PCI immediately after the failure of thrombolysis, but in the county hospitals, due to the low literacy and economic level of patients, a small number of patients refuse further interventional treatment due to symptom relief or old age. 3. Patients with indications for reperfusion therapy have a severe poor prognosis due to inadequate knowledge of the severity of the disease or delayed consultation for financial reasons [46] or refusal of any reperfusion therapy measures. 4. The overall level of a medical emergency in primary care hospitals is limited, and the advanced age of patients at the time of consultation, heart failure, and cardiogenic shock are independent risk factors for high mortality in patients with acute STEMI. The results of this study showed that a higher rate of thrombolytic therapy in patients with acute STEMI in the county hospitals during the COVID-19 epidemic did not increase the incidence of adverse events. However, in STEMI patients aged 65 and above, TT and primary PCI have similar results [47]. Patients with STEMI in Thailand had more PCI access, and PCI was linked with decreased mortality when compared to thrombolysis alone [48].

It is difficult to treat STEMI patients during a pandemic. While thrombolysis may be a useful option in some cases, many patients have contraindications, and thrombolysis would consume additional resources in these limited hospital settings. Furthermore, given the high likelihood of normal coronary arteries on coronary angiograms during acute infections and pandemics, clinicians should employ primary PCI as the first line of treatment rather than thrombolytics in these stressful conditions. To distinguish actual STEMIs from imitators, non-invasive diagnostic tests may be effective. In addition, cardiac catheterization staff must be trained on how to utilize personal protective equipment to reduce the risk of infection [49].

## 6 Limitation

This is a single-center retrospective observational study with small sample size and only in-hospital prognosis, which has limitations. Furthermore, because this trial was conducted over a long period of time in a low-risk pandemic locale, and the principles of treating STEMI were not altered, the timing of S2MFC was not dramatically altered. Although this study analyzed the comparability of the two groups by comparing age, sex, and comorbidities at baseline (all *P* > 0.05), the subjects were not randomized, so propensity score matching should be done in future studies to reduce the bias arising from the non-randomized treatment of the subjects. Another limitation is that even though none of the enrolled patients were mechanically ventilated, a detailed assessment of the patients’ respiratory impairment should be performed during the COVID-19 epidemic. Future large-scale, multicenter, and prospective clinical trials are needed to further demonstrate the impact of early PCI and direct PCI after thrombolysis on the salvage of patients with acute STEMI.

## 7 Conclusion

During the COVID-19 epidemic, the county hospitals provided thrombolytic treatment to more acute STEMI patients, but the frequency of adverse outcomes such bleeding complications, heart failure, shock, malignant arrhythmias, and death remained constant. Because emergency PCI lowered the risk of mortality in older persons, we should reconsider the optimal STEMI treatment approach and if PCI should retain the present pre-defined STEMI care strategy in rural areas.

## References

[1] China Cardiovascular Health and Disease Report Writing Group. Summary of the china cardio-vascular health and disease report 2020. Chin J Circ. 2021;36(6):521–45.

[2] Yeh RW, Sidney S, Chandra M, Sorel M, Selby JV, Go AS. Population trends in the incidence and outcomes of acute myocardial infarction. N Engl J Med. 2010;362(23):2155–65. 10.1056/NEJMoa0908610 20558366

[3] Koopman C, Bots ML, van Oeffelen AA, van Dis I, Verschuren WM, Engelfriet PM, et al. Population trends and inequalities in incidence and short-term outcome of acute myocardial infarction between 1998 and 2007. Int J Cardiol. 2013;168(2):993–8. 10.1016/j.ijcard.2012.10.036 23168007

[4] Krumholz HM, Wang Y, Chen J, Drye EE, Spertus JA, Ross JS, et al. Reduction in acute myocardial infarction mortality in the united states: risk-standardized mortality rates from 1995-2006. JAMA. 2009;302(7):767–73. 10.1001/jama.2009.1178 PMC3349070 19690309

[5] Chatterjee P, Joynt Maddox KE. US national trends in mortality from acute myocardial infarction and heart failure: policy success or failure? JAMA Cardiol. 2018;3(4):336–40. 10.1001/jamacardio.2018.0218 PMC5875358 29541764

[6] Smolina K, Wright FL, Rayner M, Goldacre MJ. Determinants of the decline in mortality from acute myocardial infarction in England between 2002 and 2010: Linked national database study. BMJ. 2012;344:d8059. 10.1136/bmj.d8059 PMC3266430 22279113

[7] Shen HM, Chen SB, Cui YB, Xu B, Kassegne K, Abe EM, et al. Geographic variations in in‐hospital mortality and use of percutaneous coronary intervention following acute myocardial infarction in China: A nationwide cross‐sectional analysis. J Am Heart Assoc. 2018;7(8):e008131.

[8] Eberhardt MS, Pamuk ER. The importance of place of residence: Examining health in rural and non-rural areas. Am J Public Health. 2004;94:1682–6. 10.2105/ajph.94.10.1682 PMC1448515 15451731

[9] Australian Institute of Health and Welfare (AIHW) Cardiovascular Disease Series. Canberra, Australia: AIHW; 2011. Cardiovascular Disease: Australian Facts 2011.

[10] Chinese Medical Association Chinese Society of Cardiology, Editorial Board of Chinese Journal of Cardiology. Guideline on diagnosis and treatment of patients with acute ST segment elevation myocardial infarction. Chin J Cardiol. 2019;47:766–83. 10.3760/cma.j.issn.0253-3758.2019.10.003 31648459

[11] Bolognese L. Treatment of ST-elevation myocardial infarction: State of the art and new horizons. Giornale italiano dicardiologia (2006). 2021;22(3):167–80. 10.1714/3557.35334 33687367

[12] Peng N, Xiao H, Dong Y, Meng Q, Zheng T, Cui X, et al. Early reperfusion strategy selection and prognosis analysis in patients with acute ST segment elevation myocardial infarction: based on the data of 49 hospitals in Hebei Province. Zhonghua Wei Zhong Bing Ji Jiu Yi Xue. 2021;33(5):578–81. 10.3760/cma.j.cn121430-20210207-00228 34112296

[13] Yang JG, Xu HY, Gao XJ, Li W, Wang Y, Li WM. Analysis of reperfusion therapy and secondary prevention medication in hospitalized patients with acute ST-segment elevation myocardial infarction in provincial, municipal and county hospitals in China. Chin J Circ. 2017;32(1):12–6.

[14] Wang YL, Jin YH, Chen HB, Lu XT, Feng XL, Dai HL. The impact of chest pain center construction on the treatment of acute myocardial infarction in primary hospitals. Chin Health Emerg Electron J. 2021;7(2):79–82.

[15] Hudzik B, Budaj A, Gierlotka M, Witkowski A, Wojakowski W, Zdrojewski T, et al. Assessment of quality of care of patients with ST-segment elevation myocardial infarction. Eur Heart J Acute Cardiovasc Care. 2020;9(8):893–901. 10.1177/2048872619882360 31762288

[16] Grines CL, Browne KF, Marco J, Rothbaum D, Stone GW, O'keefe J, et al. A comparison of immediate angioplasty with thrombolytic therapy for acute myocardial infarction. The primary angioplasty in myocardial infarction study group. N Engl J Med. 1993;328:673–9. 10.1056/NEJM199303113281001 8433725

[17] Zijlstra F, de Boer MJ, Hoorntje JC, Reiffers S, Reiber JH, Suryapranata H. A comparison of immediate coronary angioplasty with intravenous streptokinase in acute myocardial infarction. N Engl J Med. 1993;328:680–4. 10.1056/NEJM199303113281002 8433726

[18] Keeley EC, Boura JA, Grines CL. Primary angioplasty versus intravenous thrombolytic therapy for acute myocardial infarction: A quantitative review of 23 randomised trials. Lancet. 2003;361:13–20. 10.1016/S0140-6736(03)12113-7 12517460

[19] Widimský P, Budesínský T, Vorác D, Groch L, Zelízko M, Aschermann M, et al. Long distance transport for primary angioplasty vs immediate thrombolysis in acute myocardial infarction. Final results of the randomized national multicentre trial–PRAGUE-2. Eur Heart J. 2003;24:94–104. 10.1016/s0195-668x(02)00468-2 12559941

[20] Ibanez B, James S, Agewall S, Antunes MJ, Bucciarelli-Ducci C, Bueno H, et al. 2017 ESC guidelines for the management of acute myocardial infarction in patients presenting with ST-segment elevation: the task force for the management of acute myocardial infarction in patients presenting with ST-segment elevation of the European society of cardiology (ESC). Eur Heart J. 2018;39:119–77. 10.1093/eurheartj/ehx393 28886621

[21] WHO Director-General’s opening remarks at the media briefing on COVID-19-11 March, 2020 [EB/OL]. https//www.who.int/dg/speech-es/detail/who-director-general-s-opening-remarks-at-the-media-briefing-oncovid-19-11-march-2020.

[22] Announcement of the National Health Care Commission of the People’s Republic of China [EB/OL]. (2020-1-20) [2020-02-05]. http//www.nhc.gov.cn/xcs/zhengcwj/202001/44a3b8245e8049d2837a4f27529cd386.shtml.

[23] Gramegna M, Baldetti L, Beneduce A, Pannone L, Falasconi G, Calvo F, et al. ST-segment-elevation myocardial infarction during COVID-19 Pandemic: Insights from a regional public service healthcare hub. Circ Cardiovasc Interv. 2020 Aug;13(8):e009413. 10.1161/CIRCINTERVENTIONS.120.009413 32791953

[24] Pan YC, Li SN, Luo YS, Luo SL, Liu Z, Li ZC. Impact of chest pain center treatment process on the treatment of patients with ST-segment elevation myocardial infarction under the novel coronavirus pneumonia epidemic. Lingnan J Cardiovasc Dis. 2020;26(6):689–94.

[25] Fu XY, Shen XF, Cheng YR, Zhou MY, Ye L, Feng ZH, et al. Effect of COVID-19 outbreak on the treatment time of patients with acute ST-segment elevation myocardial infarction. Am J Emerg Med. 2021;44:192–7. 10.1016/j.ajem.2020.09.038 PMC7494496 33039221

[26] Primessnig U, Pieske BM, Sherif M. Increased mortality and worse cardi outcome of acute myocardial infarction during the early COVID-19 pandemic. ESC Heart Fail. 2021;8(1):333–43. 10.1002/ehf2.13075 PMC7835606 33283476

[27] Nef HM, Elsässer A, Möllmann H, Abdel-Hadi M, Bauer T, Brück M, et al. Impact of the COVID-19 pandemic on cardiovascular mortality and catherization activity during the lockdown in central Germany: An observational study. Clin Res Cardiol. 2021;110(2):292–301. 10.1007/s00392-020-01780-0 PMC7680078 33219854

[28] Saad M, Kennedy KF, Imran H, Louis DW, Shippey E, Poppas A, et al. Association between COVID-19 diagnosis and in-hospital mortality in patients hospitalized with ST-segment elevation myocardial infarction. JAMA. 2021;326(19):1940–52. 10.1001/jama.2021.18890 PMC8596198 34714327

[29] Lauridsen MD, Butt JH, Østergaard L, Møller JE, Hassager C, Gerds T, et al. Incidence of acute myocardial infarction-related cardiogenic shock during corona virus disease 19 (COVID-19) pandemic. Int J Cardiol Heart Vasc. 2020;31:100659. 10.1016/j.ijcha.2020.100659 PMC7553065 33072848

[30] Tam CF, Cheung KS, Lam S, Wong A, Yung A, Sze M, et al. Impact of coronavirus disease 2019 (COVID-19) outbreak on outcome of myocardial infarction in Hong Kong, China. Catheter Cardiovasc Interv. 2021;97:E194–7. 10.1002/ccd.28943 PMC7267252 32367683

[31] Scholz KH, Lengenfelder B, Thilo C, Jeron A, Stefanow S, Janssens U, et al. Impact of COVID-19 outbreak on regional STEMI care in Germany. Clin Res Cardiol. 2020;109:1511–21. 10.1007/s00392-020-01703-z PMC7364412 32676681

[32] Fan F, Li Y, Zhang Y, Li J, Liu J, Hao Y, et al. CCC‐ACS Investigators. Chest Pain Center Accreditation Is Associated With Improved In-Hospital Outcomes of Acute Myocardial Infarction Patients in China: Findings From the CCC-ACS Project. 2019;8(21):e013384. 10.1161/JAHA.119.013384 PMC6898834 31630594

[33] Soylu K, Coksevim M, Yanık A, Bugra Cerik I, Aksan G. Effect of Covid-19 pandemic process on STEMI patients timeline. Int J Clin Pract. 2021;75(5):e14005. 10.1111/ijcp.14005 PMC7883118 33400345

[34] Aldujeli A, Hamadeh A, Briedis K, Tecson KM, Rutland J, Krivickas Z, et al. Delays in presentation in patients with acute myocardial infarction during the COVID-19 pandemic. Cardiol Res. 2020;11(6):386–91. 10.14740/cr1175 PMC7666599 33224384

[35] Bonnet G, Panagides V, Becker M, Rivière N, Yvorel C, Deney A, et al. ST-segment elevation myocardial infarction: Management and association with prognosis during the COVID-19 pandemic in France. Arch Cardiovasc Dis. 2021;114(5):340–51. 10.1016/j.acvd.2021.01.005 PMC9056233 33926830

[36] Moroni F, Gramegna M, Ajello S, Beneduce A, Baldetti L, Vilca LM, et al. Collateral damage: Medical care avoidance behavior among patients with myocardial infarction during the COVID-19 pandemic. JACC Case Rep. 2020;2(10):1620–4. 10.1016/j.jaccas.2020.04.010 PMC7252183 32835261

[37] Baldi E, Auricchio A, Cresta R, Vanetta C, Anselmi L, Pedrazzini G, et al. Patient voluntari-ly delays call to emergency medical system for ST-elevation myocardial infarction during COVID-19 pandemic. Int J Cardiol Heart Vasc. 2021;35:100824. 10.1016/j.ijcha.2021.100824 PMC8193031 34131581

[38] Lin QH, Xu XD, Zhang YK, Wang F, Gu JH, Xu YW, et al. Reperfusion time in patients with acute ST-segment elevation myocardial infarction with different arrival modes and factors influencing outcome. Chin J Emer Med. 2020;29(7):921–8.

[39] Zhu SQ, Fan Z, Hao Y, Pu FS, Jiang GY, Yi L, et al. Impact of the new coronary pneumonia epidemic on patients with acute ST-segment elevation myocardial infarction in a tertiary care hospital in Shenzhen. Chin Electron J Health Emer. 2020;6(5):257–61.

[40] Nan J, Meng S, Hu H, Jia R, Jin Z. Fibrinolysis therapy combined with deferred pci versus primary angioplasty for STEMI patients during the COVID-19 pandemic: Preliminary results from a single center. Intern J Gen Med. 2021;14:201–9. 10.2147/IJGM.S292901 PMC7838526 33519227

[41] Leng WX, Yang JG, Li XD, Jiang WY, Gao LJ, Wu Y, et al. Impact of the shift to a fibrinolysis-first strategy on care and out-comes of patients with ST-segment-elevation myocardial infarction during the COVID-19 pan-demic-The experience from the largest cardiovascular-specific centre in China. Intern J cardiol. 2020;S0167(20):260–5. 10.1016/j.ijcard.2020.11.074 PMC7723441 33307137

[42] China Chest Pain Center Alliance, China Cardiovascular Health Alliance, China Physicians Association Chest Pain Professional Committee. Summary of the quality control report of chinese chest pain centers (2020). Chin J Interven Cardiol. 2021;29(6):313–7.

[43] Zhang H, Zheng W, Wu S, Ma JJ, Wang GM, Li Y, et al. Analysis of potential factors contributing to refusal of invasive strategy after ST-segment elevation myocardial infarction in China. Chin Med J (Engl). 2021;134(5):524–31. 10.1097/CM9.0000000000001171 PMC7929575 33652458

[44] Jacobs AK, Ali MJ, Best PJ, Bieniarz MC, Bufalino VJ, French WJ, et al. Systems of care for ST-segment-elevation myocardial infarction: A policy statement from the American Heart Association. Circulation. 2021;144(20):e310–27. 10.1161/CIR.0000000000001025 34641735

[45] Zhang Y, Tian Y, Dong IS, Xu Y, Yu B, Li H, et al. The CSCAP-1 Investigators. Treatment Delay and Reperfusion Management of Acute ST-segment Elevation Myocardial Infarction - analysis of the China STEMI Care Project Phase 1 (CSCAP-1). QJM Mon J Assoc Phys. 2020;114(5):299–305. 10.1093/qjmed/hcaa186 32569364

[46] Yin X, He Y, Zhang J, Song F, Liu J, Sun G, et al. Patient-level and system-level barriers associated with treatment delays for ST elevation myocardial infarction in China. Heart. 2020;106(19):1477–82. 10.1136/heartjnl-2020-316621 32580976

[47] Kocayigit I, Yaylaci S, Osken A, Aydın E, Sahinkus S, Can Y, et al. Comparison of effects of thrombolytic therapy and primary percutaneous coronary intervention in elderly patients with acute ST-segment elevation myocardial infarction on in-hospital, six-month, and one-year mortality. Arch Med Sci Atheroscler Dis. 2019;4:e82–8. 10.5114/amsad.2019.85378 PMC6554752 31211274

[48] Limwattananon C, Jaratpatthararoj J, Thungthong J, Limwattananon P, Kitkhuandee A. Access to reperfusion therapy and mortality outcomes in patients with ST-segment elevation myocar-dial infarction under universal health coverage in Thailand. BMC Cardiovasc Disord. 2020;20(1):121. 10.1186/s12872-020-01379-3 PMC7060593 32143572

[49] Yerasi C, Case BC, Forrestal BJ, Chezar-Azerrad C, Hashim H, Ben-Dor I, et al. Treatment of ST-segment elevation myocardial infarction during COVID-19 pandemic. Cardiovasc Revasc Med. 2020;21(8):1024–9. 10.1016/j.carrev.2020.05.027 PMC7241397 32471712

